# Robust learning framework for a scalable remote monitoring of autonomic dysreflexia: use-case in spinal cord injury

**DOI:** 10.1038/s41598-025-33797-8

**Published:** 2026-02-24

**Authors:** Bertram Fuchs, Mehdi Ejtehadi, Ana Cisnal, Jürgen Pannek, Anke Scheel-Sailer, Robert Riener, Inge Eriks-Hoogland, Diego Paez-Granados

**Affiliations:** 1 SCAI-Lab, Department of Health Sciences and Technology (D-HEST), ETH Zurich, GLC, Gloriastrasse 37/39, 8092 Zurich, Switzerland; 2 School of Computation, Information and Technology, Technical University of Munich, Arcisstraße 21, 80333 Munich, Germany; 3 Swiss Paraplegic Research (SPF), Guido A. Zäch Strasse 4, 6207 Nottwil, Switzerland; 4 Institute of Advanced Production Technologies, University of Valladolid, Paseo Prado de la Magdalena 3-5, 470011 Valladolid, Spain; 5 Swiss Paraplegic Centre (SPZ), Guido A. Zäch Strasse 1, 6207 Nottwil, Switzerland; 6 Balgrist University Hospital, Forchstrasse 340, 8008 Zurich, Switzerland

**Keywords:** Spinal cord injury, Autonomic dysregulation, Autonomic dysreflexia, Artificial intelligence, Multimodal data fusion, wearable sensors, Mobile health, Biomarkers, Computational biology and bioinformatics, Engineering, Health care, Medical research, Neuroscience

## Abstract

The autonomous nervous system (ANS) response in neurological disorders is a direct modifiable risk factor for cardiovascular health, however, difficulty in remote monitoring and objective assessment has made it underrepresented in preventive healthcare. Particularly, autonomic dysreflexia (AD) is a dangerous hypertensive emergency, potentially life-threatening in people with spinal cord injury (SCI), yet detection outside clinical settings remains reactive and episodic. This study presents an interpretable and scalable framework for creating a digital biomarker from multimodal wearables in data scarcity through vital sign attribution analysis in multiple body locations, evaluated with 27 subjects undergoing clinical examination with objective blood pressure measurements. Our framework learns from diverse biosignals—lectrocardiography (ECG), photoplethysmography (PPG), bioimpedance, skin temperature, heart rate, and respiratory rate—proving robustness to sensor failure and a pathway to remote monitoring. This study identified heart rate and ECG as dominant predictors, with PPG providing complementary value, under simulated single-modality failure or noisy channels. This work advances a feasible path to equitable, remote monitoring of the ANS response, reducing dependence on intermittent BP measurements and enabling earlier intervention.

## Introduction

Spinal cord injury (SCI) is a life-altering condition affecting approximately 52.5 individuals per million globally each year^1^, with over 58% of cases resulting in cervical-level injuries^2^. Beyond motor and sensory impairments, SCI frequently disrupts autonomic cardiovascular control, giving rise to autonomic dysregulation (ADys), a term encompassing both autonomic dysreflexia (AD) and orthostatic hypotension (OH)^3^.

AD affects up to 90% of individuals with injuries above the T6 level^4^, characterized by rapid, often severe increases in systolic blood pressure (SBP) that can result in strokes, seizures, or even death if not treated^5^. In contrast, OH involves a significant drop in blood pressure (BP) upon standing, leading to fatigue, dizziness, and reduced quality of life^6^. Both conditions contribute to elevated cardiovascular risk in SCI individuals, especially during the early months post-injury when episodes frequently begin to manifest^7^. Prompt detection and intervention are therefore essential.

Despite the clinical importance, ADys episodes often go undetected outside of hospital environments due to subtle or absent perceivable symptoms and limited awareness among caregivers^4^. Current diagnosis is primarily based on invasive monitoring or subjective patient-reported symptoms, which limits scalability and reliability in everyday settings. An objective, automated, and non-invasive detection system based on wearable sensors could enable earlier identification and timely response to ADys episodes, improving outcomes for the SCI population.

AD remains a critical and under-monitored complication for individuals with SCI, especially those with lesions above T6. Existing studies have explored both human and animal models to develop automated AD detection systems using machine learning (ML).

Suresh et al.^5^ proposed an early wearable-based system leveraging electrodermal activity (EDA), heart rate (HR), and skin temperature (Temp) sensors from a wrist-worn smartwatch. Their ML model (SVM) trained on self-reported AD events by SCI participants achieved high accuracy, but relied on a small sample of seven participants and lacked systematic cross-validation. In a follow-up study^8^, the same group extended their multimodal sensing approach, incorporating a more complex telemetry system and increasing the participant count to eleven. They reported a detection accuracy of 94.1%, emphasizing real-time application feasibility in community settings. Although lack of cross validation methods and small sample size, limits the generalizability of their findings.

Sagastibeltza et al.^9^ investigated AD onset through controlled bladder filling in a clinical environment with only five human subjects. Their approach uniquely combined physiological measurements (e.g., ECG, peripheral resistance) with hormone analysis and patient history, but it remained a preliminary proof-of-concept without wearable deployment.

Pancholi et al.^10^ proposed a novel approach using deep neural networks (DNNs) trained on skin nerve activity (SKNA) data collected from a rat model via colorectal distension to induce AD. Although their system achieved high accuracy (93.9% ± 2.5%) and low false-negative rates, the use of non-human subjects limits the clinical translatability.

Lastly, Suresh et al.^11^ conducted an in-depth analysis of feature selection techniques to optimize AD detection from physiological signals. This study focused on algorithmic refinement rather than system-level deployment, further underscoring the fragmented and exploratory state of current AD detection research.

These prior works highlight the potential of multimodal biosignal analysis, particularly using EDA and Temp; however, they also expose critical limitations: reliance on subjective ground truths, lack of temporal detection, and limited generalizability to everyday environments, with EDA and skin temperature being highly prone to environmental error. Currently, no study or method has demonstrated automated detection of AD episodes using non-invasive wearable sensor data in humans, and validated it with objective BP measurements.

In this study, we addressed this gap by investigating what wearable biosignals carry the most predictiveness for detecting AD. We hypothesize that these events are associated with unique physiological changes in each person, including alterations in Temp, EDA, heart rate variability (HRV), and respiratory rate (RR) ^5,6,12^. Therefore, a highly non-linear relationship across signals is expected and exploited using ensemble ML classifiers.

In clinical practice, early detection of AD is most critical during the first months after injury, when newly injured individuals have not yet learned to recognize symptoms or avoid triggers. In these early phases, personalized historical data are typically unavailable, making within-subject model training infeasible. For clinically realistic early detection, algorithms must therefore rely on data from other individuals and demonstrate robust cross-subject generalization. This motivated our study design, where each participant contributed a single UDS recording and model evaluation was performed in an inter-subject setting.

The contributions of this work are three-fold:

1. Identification of informative signal modalities and physiological features descriptive of AD;
2. Development of the first ensemble classifier for wearable, multi-modal AD detection based on objectively measured BP variations;
3. Design of a robust ensemble classification framework with BorutaSHAP-based feature selection, capable of maintaining performance under sensor noise or modality failure, ensuring practical applicability in real-world settings.

**Fig. 1:**
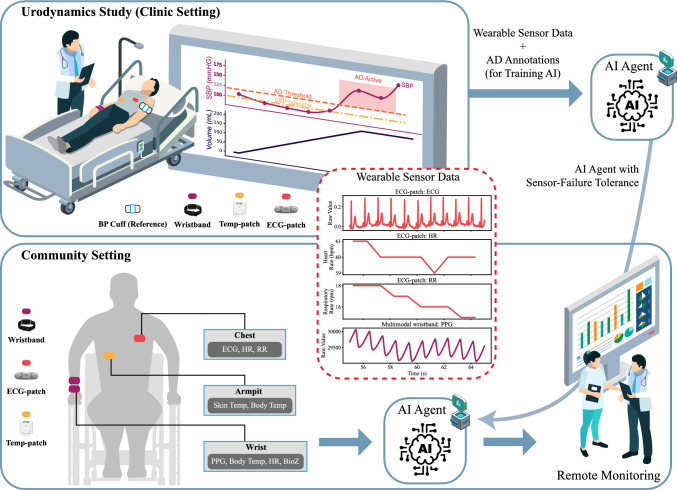
Overview of sensor setup and study framework. In the clinic setting (upper left), multimodal wristbands captured PPG, skin Temp, RR, and BioZ; a chest-mounted ECG patch recorded ECG, HR, and RR; a temperature patch measured torso Temp; and a medical cuff provided reference BP during UDS, with AD annotated by SBP rises $\ge$20 mmHg above baseline. In the community setting (lower left), the same wearable sensors were used for remote monitoring, where an AI agent trained on clinic data was deployed to detect AD episodes for further clinically actionable insights, even under sensor loss or modality failure. Exemplary wearable sensor data block (middle) illustrates representative modalities used in both settings.

An overview of our proposed framework is illustrated in Fig. 1, which depicts the wearable sensor setup, reference measurements collected in clinic, and the subsequent use of trained AI models for remote community monitoring of AD.

## Results

### Autonomic dysreflexia dataset

**Fig. 2:**
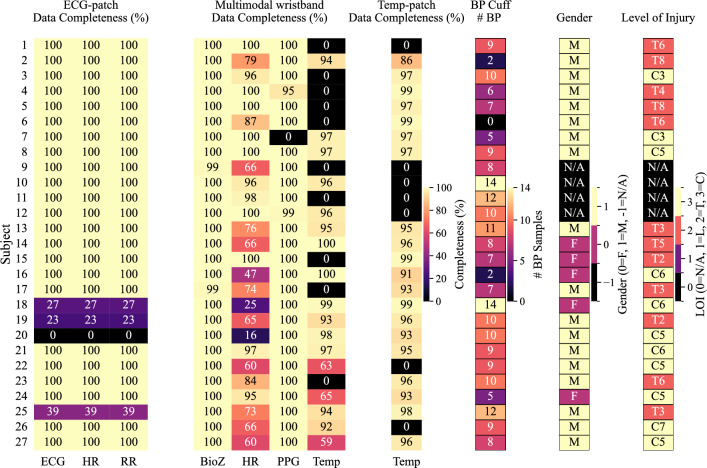
Participant overview and data completeness. The heatmap shows gender, LOI, number of reference BP samples, and sensor data completeness (%) for participants, across modalities and devices: ECG, HR, RR (ECG-patch); BioZ, HR, PPG, Temp (multimodal wristband); Temp (Temp-patch). Subjects with missing ECG/PPG or insufficient BP samples were excluded from AD classification. C, Cervical; T, Thoracic; L, Lumbar.

We first examined participant enrollment and data quality, since reliable ground truth is critical for evaluating AD detection systems. As shown in Fig. 2, 27 participants were enrolled. Gender and level of injury (LOI) data were available for 23 individuals (18 male, 5 female; 12 thoracic, 11 cervical). One thoracic case (T8) was retained despite being below the T6 threshold due to clinically confirmed AD episodes.

Ten participants were excluded from AD classification: five due to incomplete ECG or PPG data (subjects 7, 18–20, 25), three due to insufficient reference BP measurements (subjects 2, 6, 16), and two (subjects 22, 26) due to motion-induced ECG/PPG signal degradation. This resulted in 17 participants being used for model training and evaluation.

Across these 17 participants, an average of $8.58 \pm 3.01$ BP references were recorded over $21.09 \pm 6.27$ minutes of UDS. Among them, 7 exhibited AD episodes, consistent with previous reports indicating AD incidence rates of 37–78% during UDS, depending on neurological level and SCI severity^13^. Using the selected $(60\,\textrm{s},\,10\,\textrm{s})$ sliding window configuration, the final dataset included 2,145 samples (105 AD, 2,040 normal), averaging $126.2 \pm 32.2$ samples per subject, with $6.2 \pm 10.6$ ($4.6 \pm 7.6$%) labeled as AD. These figures illustrate the class imbalance typical of AD detection tasks.

### Window size analysis

**Fig. 3:**
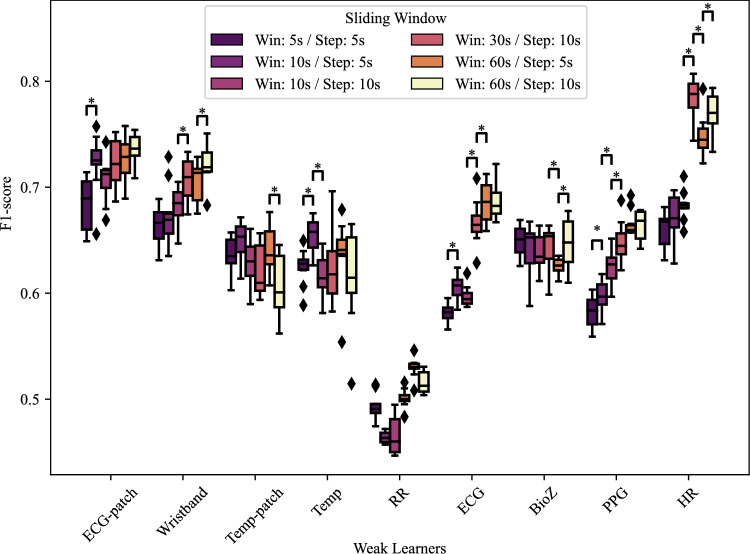
F1-score of individual weak learners (by modality and device) across different sliding window configurations. Asterisks (*) indicate statistically significant differences between consecutive window configurations (paired t-test, $p<0.05$).

Because window size directly influences the ability to capture physiological events, we compared six different configurations. Figure 3 shows the F1-score per weak learner and configuration. To assess overall performance, we also averaged F1-scores across all weak learners for different windowing configurations (duration, step): $0.62 \pm 0.12$ for (5 s, 5 s), $0.65 \pm 0.12$ for (10 s, 5 s), $0.62 \pm 0.12$ for (10 s, 10 s), $0.67 \pm 0.12$ for (30 s, 10 s), $0.67 \pm 0.12$ for (60 s, 5 s), and $0.68 \pm 0.12$ for (60 s, 10 s). Based on this analysis, (60 s, 10 s) was selected as the optimal configuration and is used in the subsequent analyses. Longer windows therefore appear to capture AD-related patterns more reliably, consistent with the clinical presentation of sustained cardiovascular changes during episodes.

### Feature importance and feature selection

To evaluate which physiological signals most strongly contribute to AD detection, we examined feature importances using both SHAP and BorutaSHAP.

**Fig. 4:**
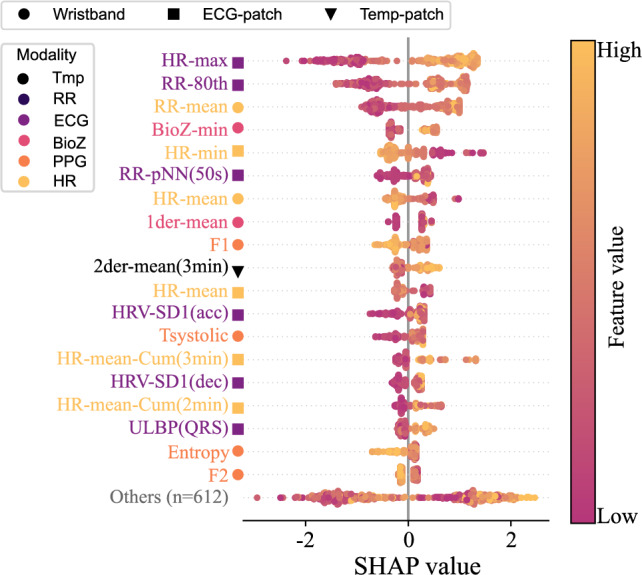
SHAP summary plot of the top features for AD detection using an XGBoost classifier trained on the original imbalanced dataset. Features are ranked by their mean absolute Shapley values. Text colors indicate signal modality; markers indicate device.

Figure 4 ranks features by Shapley importance, aiding physiological interpretation. ECG- and HR-derived features dominated, including HR statistics (mean, min), cumulative mean HR (*HR-mean-Cum*), and HRV metrics (*RR-pNN(50s)*, *RR-80th*, *RR_maxNN*). Morphological ECG features such as *ULBP(QRS)* (a QRS complexity descriptor based on Uniform Local Binary Patterns) also appeared. BioZ contributed with SCL *max* and *mean*, while PPG yielded *F1* (fundamental spectral frequency). No Temp- or RR-derived features ranked among the top, suggesting limited relevance of these modalities for classification in this dataset.

**Fig. 5:**
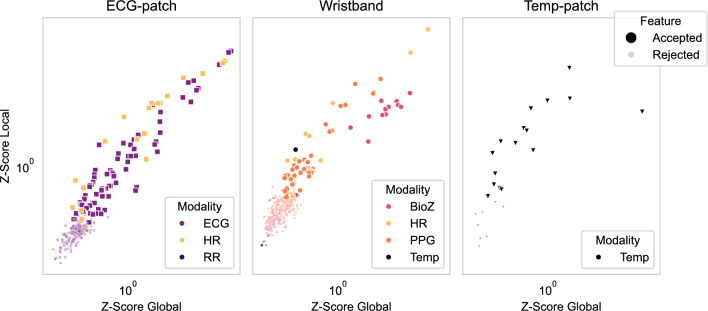
Device-wise feature importances (z-score) from BorutaSHAP. Each point is one feature. Modalities are illustrated in different colors. Accepted features are shown with big circles while rejected features are shown with smaller circles.

To complement the SHAP analysis, we applied BorutaSHAP to assess feature relevance at both device and modality levels. Figure 5 shows global (horizontal) versus local (vertical) z-scores of TreeSHAP importances, stratified by device. Global scores reflect feature relevance across all modalities, while local scores capture relevance within each device. Features in the upper-right quadrant are informative both generally and device-specifically. The multimodal wristband produced the highest number of high-scoring features; the ECG-patch contributed predominantly ECG and HR features; and the Temp-patch offered fewer but consistent Temp features.

**Fig. 6:**
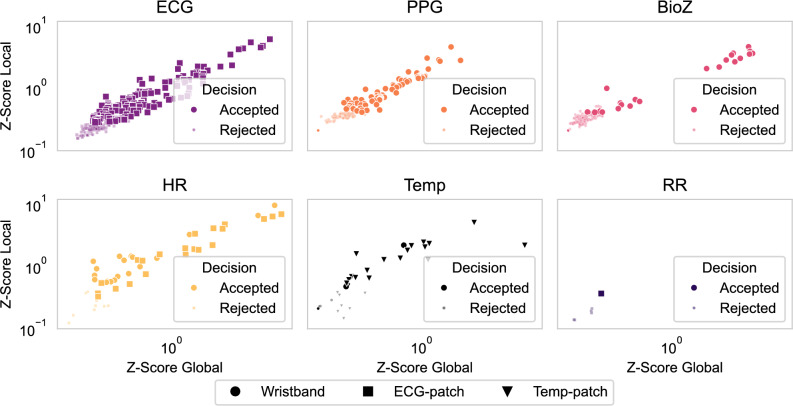
Modality-wise feature importances (z-score) from BorutaSHAP. Accepted features are shown as larger markers, while rejected features are shown as smaller markers. Marker forms indicate device.

Figure 6 presents the same analysis grouped by modality. ECG and HR yielded the most accepted features with high global and local z-scores. BioZ and PPG also contributed several discriminative features, while Temp and RR had fewer overall. Among Temp features, those from the Temp-patch device ranked higher than those from the wristband, consistent with the Temp-patch’s ability to measure both core and skin temperature. For HR, more accepted features originated from the ECG-patch than the wristband, reflecting the greater reliability of ECG-derived HR compared to PPG-derived HR.

### Learning framework through ensemble classifiers

**Fig. 7:**
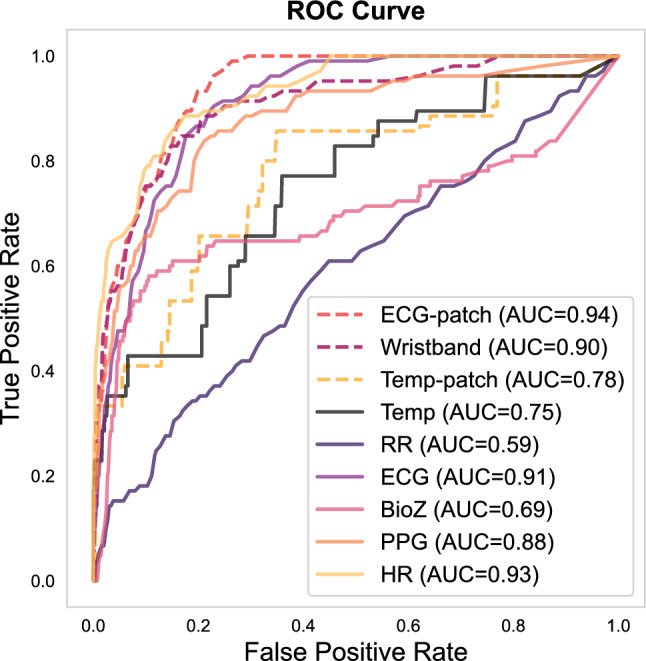
Receiver Operating Characteristic (ROC) curves of weak learners trained on individual signal modalities (Temp, RR, ECG, BioZ, PPG, HR) or device-specific feature sets (ECG-patch, multimodal wristband, Temp-patch). Curves correspond to a single run from the 10 repeated evaluations.

We then evaluated classification performance at the device, modality, and ensemble levels. Figure 7 compares ROC curves for each weak learner. Among devices, the ECG-patch achieved the highest AUC (0.94), followed by the multimodal wristband (0.90) and the Temp-patch (0.78). When analyzed by modality, HR yielded the highest AUC (0.93), closely followed by ECG (0.91) and PPG (0.88). Temp achieved a moderate AUC (0.75), while BioZ and RR had lower predictive values (0.69 and 0.59, respectively). These results highlight the central role of cardiac-derived signals in AD detection, while Temp, BioZ, and RR were less discriminative.

**Fig. 8:**
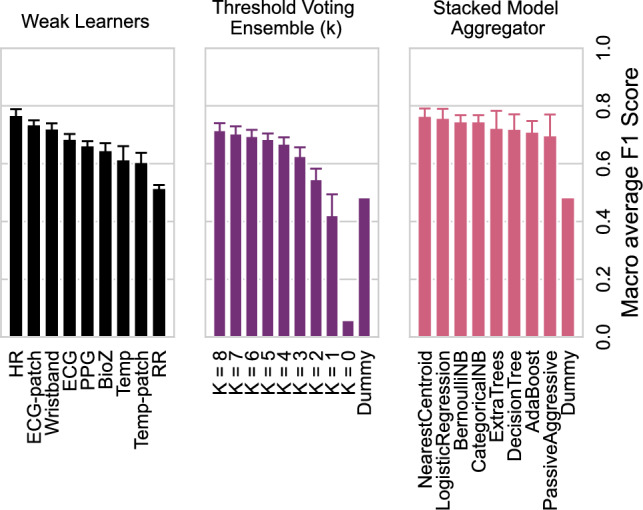
Macro F1-scores of individual weak learners (left), *k*-threshold voting ensembles (middle), and stacked model aggregators (right). Learners include both modality- and device-specific models. Ensemble voting is shown across *k* thresholds. The Dummy classifier serves as a baseline.

Beyond AUC, we examined macro F1-scores for all model components (Fig. 8). Individual weak learners confirmed the strong performance of HR and ECG-patch models. Ensemble strategies improved results further: *k*-threshold voting achieved the highest performance at $k=8$ (0.72 ± 0.02), while stacked aggregators outperformed both individual learners and threshold ensembles, with Nearest Centroid achieving the top F1-score (0.77 ± 0.03). A Dummy classifier yielded 0.48 ± 0.00 as a baseline. These results confirm that ensemble frameworks enhance robustness and stability in AD detection.

**Fig. 9:**
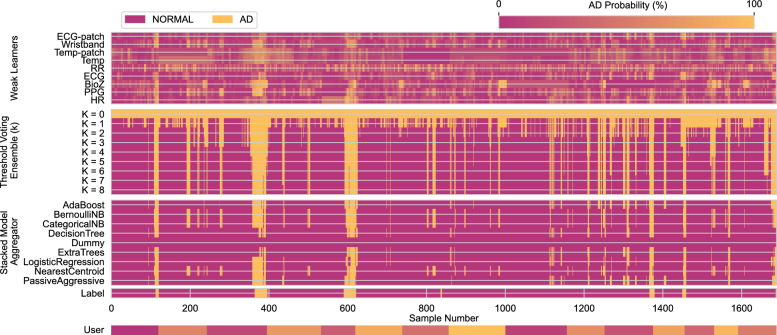
Temporal prediction overview across all samples of the dataset. Rows show AD prediction probabilities from: individual weak learners, *k*-Threshold Voting Ensembles ($k=0$ to $k=8$), stacked model aggregators, user IDs, and ground truth labels. Samples are ordered by subject and time. A Dummy classifier is included for baseline comparison.

We also visualized prediction dynamics across subjects (Fig. 9). The top panel shows probabilistic outputs of individual weak learners, the middle panel illustrates *k*-Threshold Voting Ensembles ($k=0$ to $k=8$), and the bottom panel shows stacked model aggregators, alongside user IDs and ground truth labels. This overview supports inspection of consistency, agreement, and alignment with AD episodes, enabling qualitative assessment of detection performance in terms of true detections, missed events, false positives, and early warnings.

Finally, we tested robustness under sensor limitations by evaluating all weak learner subsets (Table 1). The best subset excluded Temp and achieved an F1-score of 0.78, slightly outperforming the full 9-learner configuration (F1 = 0.77). Several other subsets performed comparably (F1 = 0.75–0.77), indicating resilience to sensor loss. HR, ECG, and BioZ consistently appeared in top-performing subsets, while PPG and Temp were more often excluded. These findings suggest that stable AD detection can be achieved even when some signals are unavailable, with HR and ECG providing the strongest backbone.

**Table 1:** Classification metrics for the top-performing weak-learner subsets. The full ensemble, comprising all nine weak learners, attains the highest F1-score. Weak learners correspond to customized classifiers trained either on (i) a single device (including all modalities available on that device) or (ii) a single modality aggregated across different sensor locations.

Weak learners	Acc	Prec	Rec	F1
All	0.93	0.73	0.85	0.77
ECG-patch, ECG, BioZ, PPG, HR, Temp-patch	0.92	0.71	0.82	0.78
Wristband, ECG, BioZ, HR, Temp-patch	0.91	0.70	0.80	0.77
Wristband, ECG, HR, Temp-patch, Temp	0.91	0.70	0.78	0.76
ECG-patch, ECG, BioZ, HR, Temp-patch	0.91	0.70	0.79	0.76
ECG, PPG, HR, Temp-patch	0.89	0.68	0.76	0.75

## Discussion

This study demonstrates the feasibility of detecting AD in individuals with SCI, using non-invasive wearable sensors. AD remains a critical clinical concern because of its unpredictable onset, silent presentation in some patients, and potential to trigger life-threatening complications such as stroke. Our findings show that a multimodal wearable approach, combined with ensemble ML methods, can identify AD episodes with clinically meaningful accuracy. Importantly, ECG- and HR-derived features consistently emerged as the most robust predictors, supporting their role as primary markers for wearable-based AD monitoring.

The dataset (Fig. 2) revealed important considerations for real-world implementation. ECG-based HR from the ECG-patch proved more reliable than PPG-derived HR from the wristband, which was more prone to motion artifacts and signal dropout. Similarly, Temp data from the Temp-patch were more consistent than wristband-based Temp, highlighting the advantage of dedicated sensors for physiological monitoring. While BioZ and RR were generally well recorded, their predictive value was lower, consistent with ensemble analyses showing weaker contributions. These differences underscore the need for careful sensor selection in multimodal systems, particularly when moving toward ambulatory deployment where robustness is essential.

Feature-importance analyses (Figs. 4, 5 and 6) provided further insight into the physiological basis of AD detection. ECG and HR dominated the top-ranked features, including HR variability metrics, morphological ECG descriptors, and HR statistics, all of which align with established clinical observations linking cardiac dysregulation to AD episodes^12,14,15^. PPG features such as systolic timing also contributed, reflecting hemodynamic changes correlated with BP^16^. BioZ features captured aspects of sympathetic arousal. Temp features showed lower global importance, likely due to missing data (Fig. 2) and slower thermal dynamics, yet their local relevance indicates that Temp provides physiologically meaningful information for AD. Notably, RR contributed little, diverging from some earlier reports^17^. This discrepancy likely reflects differences in data-collection, as our data were recorded during UDS, whereas prior work relied on self-reported AD events. Together, these findings suggest that while cardiac signals are most informative, complementary contributions from PPG, BioZ, and Temp may provide additional discriminatory power in ensemble designs.

Temporal windowing influenced performance in clinically interpretable ways. As shown in Fig. 3, longer windows (notably 60 s) improved overall classification performance, likely by capturing more complete AD episodes and reducing imbalance between AD and normal samples. At the same time, optimal window size varied by modality: shorter durations favored Temp and RR, intermediate windows benefited BioZ, and longer ones improved ECG, HR, and PPG by capturing rhythm- and morphology-related features. These findings suggest that future detection pipelines may benefit from modality-specific windowing strategies rather than a single uniform approach.

Performance analyses confirmed the clinical utility of the proposed framework and, importantly, add device-level nuance. The ROC analysis (Fig. 7) highlighted the ECG-patch as the best-performing device (AUC = 0.94), followed by the multimodal wristband (AUC = 0.90), while the Temp-patch lagged (AUC = 0.78), likely reflecting its reliance on a single, less-informative modality (Temp), consistent with SHAP results. When analyzed by modality, HR achieved the highest AUC (0.93), followed by ECG (0.91) and PPG (0.88); Temp and BioZ were moderate (0.75 and 0.69, respectively), and RR showed the weakest performance (0.59), in line with BorutaSHAP findings (Fig. 6). According to established interpretive guidelines^18,19^, these values indicate outstanding discrimination (AUC $>0.90$) for HR and the ECG-patch device, and excellent performance (AUC 0.8–0.9) for ECG, PPG, and the wristband—supporting their clinical relevance for AD detection.

Ensemble modeling further improved robustness. The Nearest Centroid meta-classifier achieved a macro F1-score of $0.77\pm 0.03$, substantially outperforming a Dummy baseline ($0.48\pm 0.00$) (Fig. 8). Notably, the highest F1-score (0.78) was obtained with a reduced subset excluding less reliable signals (Table 1), suggesting that redundancy may dilute performance and that carefully chosen modality/device subsets can maintain or even enhance accuracy. Temporal prediction maps (Fig. 9) provided clinically relevant insights: HR and ECG aligned closely with true AD episodes, while Temp often rose earlier, potentially offering early-warning capabilities. Such dynamics highlight the complementary roles of modalities—high-specificity cardiac features for reliable detection and slower-reacting thermal features for anticipatory intervention.

Despite these encouraging results, several limitations must be acknowledged. A first limitation lies in defining reliable BP baselines for AD detection in SCI, given the high physiological variability reported in this population. Mean arterial pressure (MAP) variability while sitting has been measured at $17\pm 4$ mmHg (20% MAP) in tetraplegia and $13\pm 2$ mmHg (12%) in paraplegia; in recumbency, variability was $13\pm 3$ mmHg (20%) and $8\pm 2$ mmHg (8%), respectively^20^. We adopted the practical baseline definition from^21^, averaging the first three resting BP values before UDS, but this may not capture circadian or lesion-specific variability, which could affect labeling accuracy^22^. Sparse BP sampling also required interpolation, potentially distorting onset and duration of AD labeling. In addition, although PPG availability was nearly complete for all participants (Fig. 2), the derived HR exhibited substantial missingness, highlighting the vulnerability of secondary metrics. While the Corsano device has shown good performance in controlled studies (with an accuracy of 3 beats per minute^23^), such performance does not necessarily translate to our UDS with SCI population. Furthermore, the modest sample size (27 participants, 17 retained, 7 with AD episodes) limits generalizability, underscoring the need for larger multicenter datasets. Finally, our analyses were conducted during controlled UDS; translation to ambulatory environments remains an open challenge given motion artifacts and uncontrolled conditions. In such real-world conditions, dedicated motion-artifact detection methods, extending beyond the SQI filters applied in this study, will be essential to ensure reliable AD episode detection.

Compared with prior work, our study advances the field in several respects. Earlier investigations relied heavily on self-reports^5,8^, small pilot samples^9^, or animal models^10^, limiting generalizability. Others explored algorithmic strategies without integrating them into clinically validated pipelines^11^. By contrast, our work applies stratified cross-validation, uses objective BP-based ground-truth labeling, and evaluates performance directly in human SCI participants under real clinical conditions. These methodological choices enhance both physiological validity and clinical relevance. Recent multimodal wearable studies further underscore this potential. For example, Berkebile et al. used wearable multimodal sensing to quantify the cardiovascular autonomic responses to levodopa in parkinsonism^24^. Similarly, Qiu et al. introduced a stroke-volume-allocation model enabling wearable sensors to estimate vascular age and cardiovascular disease risk^25^. Together, these developments situate our AD-focused framework within a broader class of emerging, interpretable, multi-parameter cardiovascular monitoring systems.

In summary, this study provides a proof-of-concept for wearable-based AD detection using multimodal signals, with ECG- and HR-derived features emerging as the most reliable indicators. While limitations remain—particularly regarding sample size and generalizability—the results point to a viable pathway toward non-invasive, scalable AD monitoring systems. Future efforts should focus on larger datasets, ambulatory validation, and integration of early-warning capabilities. Ultimately, such systems could enable timely intervention, reduce the risk of hypertensive crises, and improve the quality of life for individuals living with SCI.

## Conclusions

This study successfully developed and validated a robust multimodal wearable-sensor system for automated, non-invasive AD detection in individuals with SCI, addressing a critical need for personalized cardiovascular monitoring. Our findings underscore the significant role of HR and ECG in accurately identifying AD episodes, providing reliable physiological markers for this life-threatening condition. The ensemble classification framework demonstrated resilience to sensor limitations, with key modalities maintaining strong predictive performance, which is vital for real-world application. This work represents a crucial step toward empowering individuals with SCI through continuous, personalized health surveillance, enabling earlier detection and proactive management of AD events. Future efforts will focus on optimizing these systems for everyday ambulatory use, further enhancing personalized health-care and preventive interventions.

## Methods

### Study population

This observational proof-of-concept study included 27 individuals with chronic, motor- and sensory-complete SCI (AIS A) with level of injury (LOI) at or above the T6 level. Participants were recruited from inpatient and outpatient services at the Swiss Paraplegic Center (SPC), where they underwent a scheduled urodynamic study (UDS). Data were compiled from three ethically approved research protocols.

The compiled dataset was screened for completeness, and subjects with insufficient ECG, PPG, or reference BP measurements were excluded from analysis. Demographic characteristics and sensor data availability after screening are presented in “Results”.

### Clinical protocol and reference AD labels

During UDS, the bladder was filled with 37°C saline solution to induce AD. SBP, DBP, and HR were recorded every 2–3 min using a medical-grade BP cuff. Reference AD episodes were annotated using clinical guidelines from Krassioukov et al.^3^ and the American Autonomic Society^26^, which define AD as a sustained increase in SBP of $\ge$ 20 mmHg above baseline. The baseline was computed as the average of the first three SBP measurements obtained during the UDS. A piecewise-cubic Hermite interpolating polynomial (PCHIP) was fitted to the sparse BP data to generate continuous reference labels. Figure 1 illustrates a representative AD episode with interpolated SBP, AD threshold, and bladder filling.

### Wearable sensor system

A multimodal wearable system recorded photoplethysmography (PPG), electrocardiography (ECG), bio-impedance (BioZ), skin and core body temperature (collectively referred to as Temp), HR, and respiratory rate (RR). Two multimodal wristbands (CardioWatch, Corsano) captured PPG, skin temperature, HR, and BioZ. A chest-mounted ECG-patch (Wearable ECG Monitor, VivaLNK) recorded ECG, HR, and RR. A Temp-patch (CORE, greenTEG) provided both skin and core body temperature. Table 2 summarizes device specifications, and Fig.  1 shows device placement. Both Corsano wristbands were positioned on the right wrist, while the BP cuff was placed on the left arm to avoid interference between cuff inflation and wearable recordings, following protocols used in previous studies^27^.

**Table 2:** Sensory setup and device specifications used for the classification of AD.

Device ID	Device model	Modalities
Multimodal wristband	CardioWatch 287-2B (Corsano Health Inc., Cambridge, MA, USA)	PPG (128 Hz), Temp (1/60 Hz), RR (1 Hz), BioZ (32 Hz)
ECG-patch	Wearable ECG monitor (VivaLNK, Campbell, CA, USA)	ECG (128 Hz), HR (1 Hz), RR (1 Hz)
Temp-patch	CORE (greenTEG, Zurich, CH)	Temp (1/60 Hz)

### Pre-processing and feature extraction

Signals were synchronized across devices, denoised, and quality-controlled as needed. All sensors were connected to the same phone device, ensuring that their internal clocks were initially aligned. To correct for potential drift caused by inaccuracies in the individual sensor clocks, synchronization events were performed at both the beginning and the end of the measurement period. Each synchronization event consisted of a distinctive motion pattern that was clearly identifiable in the accelerometer data of all devices. The corresponding reference timestamps for these events were also recorded. The sensor time series were then linearly stretched or compressed to align them with one another and with the reference timeline.

Each biosignal underwent modality-specific preprocessing and feature extraction using sliding windows with (duration, step) configurations: (5 s, 5 s), (10 s, 5 s), (10 s, 10 s), (30 s, 10 s), (60 s, 5 s), and (60 s, 10 s) Features were computed per window to capture patterns relevant to AD detection. For ECG and PPG, additional template-based features were extracted from beat-to-beat waveforms, resulting in both window-level and beat-level descriptors. Table 3 summarizes preprocessing and feature types per modality. The final set includes time-domain, statistical, and frequency-domain features, derived using custom methods and open-source libraries (VitalPy^28^, NeuroKit2^29^, BIOBSS^30^).

**Table 3:** Summary of preprocessing and feature extraction per modality. AI, Augmentation Index; CT, Crest Time; DW, Diastolic Width; FFT, Fast Fourier Transform; HRV, Heart Rate Variability; LASI, Large Artery Stiffness Index; LBP, Local Binary Patterns; LF/HF, Low-/High-Frequency ratio; MAD, Median Absolute Deviation; NPV, Normalized Pulse Volume; pNN50, Proportion of NN intervals $>50\,\text {ms}$; RI, Reflection Index; RMSSD, Root Mean Square of Successive Differences; RMS, Root Mean Square; SCL, Skin Conductance Level; SCR, Skin Conductance Response; SDNN, Standard Deviation of NN intervals; SNR, Signal-to-Noise Ratio; SW, Systolic Width.

Modality	Preprocessing	Features extracted
PPG	Signal inversion (sensor reflection correction), 4th-order Butterworth bandpass (0.25–10 Hz), baseline correction via iteratively reweighted least squares^31^, heartbeat segmentation with derivative-based adaptive thresholding^28^, systolic peak normalization, and SQI filtering (skewness, kurtosis, SNR) with subject-specific thresholds	Template-based features: fiducial timing, amplitudes, slopes, areas, and indices (RI, AI, CT, NPV, LASI); statistical: mean, variance, MAD, RMS, skewness, kurtosis, entropy, perfusion; frequency: FFT harmonics, spectral stats (e.g., skewness, energy, wavelet entropy); additional temporal/spectral descriptors from VitalPy^28^ and BIOBSS^30^, including DW, SW, DW/SW ratios, amplitude percentiles, and peak FFT amplitudes
ECG	Wavelet denoising (bior4.4), sequential median filtering (200/600 ms) to correct P/QRS/T baseline drift^32^; R-peak detection using Christov’s method^33^; outlier beats removed via morphology and amplitude thresholds from baseline	Template-based: fiducial durations (QRSw, QSd), amplitude differences (PQa, QRa, RSa), amplitude ratios^34^; morphology descriptors: wavelets (db3), Hermite coefficients, LBP patterns^35^; time-series: HRV (mean HR, SDNN, RMSSD, pNN50), frequency (LF/HF via Welch), Poincaré (SD1, SD2)^29^
BioZ	Median filter (4 s) to separate tonic (SCL) and phasic (SCR) components^29^	Separate features for tonic (SCL) and phasic (SCR) components: statistical (mean, std, min/max, derivatives), SCR descriptors (peak count, amplitude, rise time, area)^36^; bandpower (energy, variance, power in 0–0.5 Hz bands); spectral (magnitude area, freq. mean/std, range, skewness, kurtosis)
Temp	None (low sampling rate)	Mean absolute value; 1st–3rd derivatives over 1-, 2-, and 3-min intervals to capture temporal trends^37^. Missing features are replaced by propagating the last valid feature
HR	Normalized to resting; missing values linearly interpolated	Time-domain features (e.g. mean RR, SDNN, RMSSD, pNN50); frequency-domain features using cubic interpolation at 4 Hz (e.g. LF, HF, LF/HF, total power); nonlinear features (Poincaré SD1, SD2)
RR	Normalized to resting	Same as Temp

### Machine learning framework

#### SHAP-based feature importance and selection

Feature importance and selection were addressed jointly using TreeSHAP^38^ and BorutaSHAP^39^. TreeSHAP computes exact per-sample Shapley values $\phi _i^{(j)}$ from XGBoost ensembles, representing the marginal contribution of feature $i$ to the prediction $f(x^{(j)})$ for sample $j$, relative to the expected model output $\mathbb {E}[f(x)]$. Global rankings used mean absolute SHAP values, z-scored across features; local relevance used standardized distributions of $\phi _i^{(j)}$.

For selection, raw features were first z-normalized. Then SHAP values were standardized:

$$\bar{\phi }_i = \frac{1}{N} \sum _{j=1}^N \phi _i^{(j)}, \quad s_i = \sqrt{ \frac{1}{N-1} \sum _{j=1}^N \left( \phi _i^{(j)} - \bar{\phi }_i\right) ^2 }, \quad z_i^{(j)} = \frac{\phi _i^{(j)} - \bar{\phi }_i}{s_i}$$

BorutaSHAP compared these standardized SHAP scores against “shadow” (permuted) features, retaining only features that performed consistently better than the shadow features. Selection was conducted both locally (per modality/device) and globally. XGBoost with column subsampling mitigated multicollinearity. Tentative features were treated as rejected after 500 iterations. To reduce label noise, only samples within 2 minutes of a reference BP measurement were considered.

#### Ensemble architecture and evaluation

To ensure robust AD detection under real-world conditions (e.g., sensor dropout or signal degradation), we implemented a multi-modal ensemble framework. The architecture comprises multiple Random Forest weak learners, each trained on features from one modality (e.g., ECG, PPG, BioZ) or device (e.g., ECG-patch, multimodal wristband, Temp-patch). Inputs were standardized via a RobustScaler and limited to features selected by BorutaSHAP. Each learner was trained on class-balanced data via random undersampling of the majority class.

Two aggregation strategies were tested: (1) *k*-threshold voting, which predicts AD if at least $k$ weak learners concur; and (2) stacked ensemble learning, where outputs from weak learners were used to train over 40 classifiers (e.g., Random Forest, XGBoost, Logistic Regression).

**Fig. 10:**
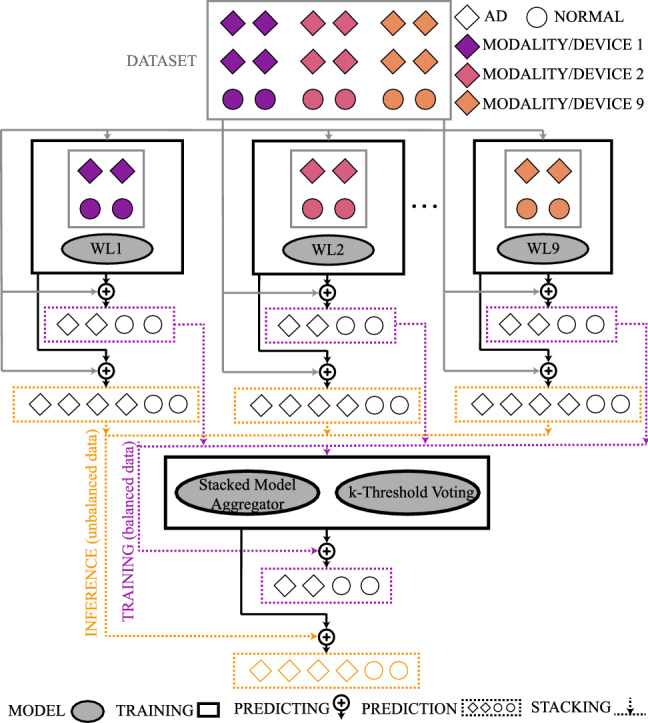
Ensemble classifier. Each of the 9 weak learners (WLi) is trained on features from a single one of the 6 modalities or 3 devices. Final predictions are aggregated via (1) *k*-threshold voting or (2) a stacked ensemble meta-learner. Training uses balanced data; validation is carried out on imbalanced sets.

Model generalization was assessed using Leave-One-Subject-Out cross-validation. Evaluation on held-out subjects used the original class imbalance to reflect clinical prevalence. To address sampling variability, training and testing were repeated 10 times with different random seeds. Figure 10 demonstrates the ensemble classifier.

Performance was evaluated via ROC-AUC and macro F1-score, accounting for class imbalance. Class-wise precision, recall, and F1 were also calculated and macro averaged.

## Data Availability

The datasets generated and analyzed during the current study are not publicly available due to participant privacy and institutional ethical restrictions. Data access may be considered upon reasonable request to the corresponding author and with appropriate ethical approvals.

## Code availability

The code used for data processing and analysis is available from the corresponding authors upon reasonable request.

## References

[1] Price, M.J. & Trbovich, M. *Thermoregulation Following Spinal Cord Injury*, vol. 157, 799–820. 10.1016/B978-0-444-64074-1.00050-1 (Elsevier, 2018). 10.1016/B978-0-444-64074-1.00050-1 30459042

[2] Singh, A., Tetreault, L., Kalsi-Ryan, S., Nouri, A. & Fehlings, M. G. Global prevalence and incidence of traumatic spinal cord injury. *Clin. Epidemiol.* **6**, 309–331. 10.2147/CLEP.S68889 (2014). 25278785 10.2147/CLEP.S68889 PMC4179833

[3] Krassioukov, A. V. et al. Assessment of autonomic dysfunction following spinal cord injury: rationale for additions to international standards for neurological assessment. *J. Rehabil. Res. Dev.* **44**(1), 103. 10.1682/JRRD.2005.10.0159 (2007). 17551864 10.1682/jrrd.2005.10.0159

[4] Allen, K. J. & Leslie, S. W. *Autonomic Dysreflexia* (StatPearls Publishing, Treasure Island, 2022). 29494041

[5] Suresh, S. & Duerstock, B. S. Automated detection of symptomatic autonomic dysreflexia through multimodal sensing. *IEEE J. Transl. Eng. Health Med.* **8**, 1–8. 10.1109/JTEHM.2019.2955947 (2020). 10.1109/JTEHM.2019.2955947 PMC7028437 32082953

[6] Bradley, J. G. & Davis, K. A. Orthostatic hypotension. *Am. Fam. Physician* **68**(12), 2393–2398 (2003). 14705758

[7] Lindan, R., Joiner, E., Freehafer, A. A. & Hazel, C. Incidence and clinical features of autonomic dysreflexia in patients with spinal cord injury. *Spinal Cord* **18**(5), 285–292. 10.1038/sc.1980.51 (1980). 10.1038/sc.1980.51 7443280

[8] Suresh, S., Everett, T. H., Shi, R. & Duerstock, B. S. Automatic detection and characterization of autonomic dysreflexia using multi-modal non-invasive sensing and neural networks. *Neurotrauma Rep.* **3**(1), 501–510. 10.1089/neur.2022.0041 (2022). 36479362 10.1089/neur.2022.0041 PMC9718431

[9] Sagastibeltza, N., Salazar-Ramirez, A., Yera, A., Martinez, R., Muguerza, J., Sanchez, N.C. & Gil, M.A.A. Preliminary study on the detection of autonomic dysreflexia using machine learning techniques. In *Advances and Applications in Computer Science, Electronics, and Industrial Engineering*, vol. 433 (eds. Garcia, M. V., Fernández-Peña, F., Gordón-Gallegos, C.) 341–351 (Springer, 2022). 10.1007/978-3-030-97719-1_20.

[10] Pancholi, S., Everett, T. H. & Duerstock, B. S. Advancing spinal cord injury care through non-invasive autonomic dysreflexia detection with AI. *Sci. Rep.* **14**(1), 3439. 10.1038/s41598-024-53718-5 (2024). 38341453 10.1038/s41598-024-53718-5 PMC10858945

[11] Suresh, S., Newton, D. T., Everett, T. H., Lin, G. & Duerstock, B. S. Feature selection techniques for a machine learning model to detect autonomic dysreflexia. *Front. Neuroinform.* **16**, 901428. 10.3389/fninf.2022.901428 (2022). 36033642 10.3389/fninf.2022.901428 PMC9416695

[12] Calderón-Juárez, M. et al. Heart rate variability-based prediction of autonomic dysreflexia after spinal cord injury. *J. Neurotrauma* **41**(9–10), 1172–1180. 10.1089/neu.2023.0583 (2024). 38214089 10.1089/neu.2023.0583

[13] Krassioukov, A. V. & Walter, M. Autonomic dysreflexia in neuro-urological practice. In *Handbook of Neurourology: Theory and Practice* (eds Liao, L. & Madersbacher, H.) 663–671 (Springer, Singapore, 2023). 10.1007/978-981-99-1659-7_81.

[14] Yee, B., Nightingale, T. E., Ramirez, A. L., Walter, M. & Krassioukov, A. V. Heart rate changes associated with autonomic dysreflexia in daily life of individuals with chronic spinal cord injury. *Spinal Cord* **60**(11), 1030–1036. 10.1038/s41393-022-00820-y (2022). 35680988 10.1038/s41393-022-00820-y

[15] Lindan, R., Joiner, E., Freehafer, A. A. & Hazel, C. Incidence and clinical features of autonomic dysreflexia in patients with spinal cord injury. *Paraplegia* **18**(5), 285–292 (1980). 7443280 10.1038/sc.1980.51

[16] Park, J., Seok, H.S., Kim, S.-S. & Shin, H. Photoplethysmogram analysis and applications: an integrative review. *Front. Physiol.* **12**. 10.3389/fphys.2021.808451 (2022). 10.3389/fphys.2021.808451 PMC8920970 35300400

[17] Suresh, S. & Duerstock, B.S. Optimal feature selection for the detection of autonomic dysreflexia in individuals with tetraplegia. In *2018 IEEE International Symposium on Signal Processing and Information Technology (ISSPIT)*, 480–485. 10.1109/ISSPIT.2018.8642624 (2018).

[18] Hosmer, D.W., Lemeshow, S. & Sturdivant, R.X. *5. Assessing the Fit of the Model* 153–225 (Wiley, 2013). 10.1002/9781118548387.ch5.

[19] Mandrekar, J. N. Receiver operating characteristic curve in diagnostic test assessment. *J. Thorac. Oncol.* **5**(9), 1315–1316 (2010). 20736804 10.1097/JTO.0b013e3181ec173d

[20] Frisbie, J. H. Unstable baseline blood pressure in chronic tetraplegia. *Spinal Cord* **45**(1), 92–95. 10.1038/sj.sc.3101920 (2007). 16568144 10.1038/sj.sc.3101920

[21] Hubli, M., Gee, C. M. & Krassioukov, A. V. Refined assessment of blood pressure instability after spinal cord injury. *Am. J. Hypertens.* **28**(2), 173–181. 10.1093/ajh/hpu122 (2014). 24990527 10.1093/ajh/hpu122

[22] Deutges, M. & Redtel, H. Autonomous calibration of blood pressure dependent data using second-order blood pressure variation for a future mobile diagnostic: requirements for a calibration. *IEEE Access* **12**, 97269–97279. 10.1109/ACCESS.2024.3426984 (2024).

[23] Food, U. S. & Administration, D. *510(k) premarket notification: Corsano cardiowatch 287–2 system (K232548)* (Technical report, Food and Drug Administration, Silver Spring, MD, 2024).

[24] Berkebile, J. A., Inan, O. T. & Beach, P. A. Wearable multimodal sensing for quantifying the cardiovascular autonomic effects of levodopa in parkinsonism. *Front. Netw. Physiol.* **5**, 1543838. 10.3389/fnetp.2025.1543838 (2025). 40342690 10.3389/fnetp.2025.1543838 PMC12058781

[25] Qiu, S., Yan, B.P. & Zhao, N. Stroke-volume-allocation model enabling wearable sensors for vascular age and cardiovascular disease assessment. *NPJ Flex. Electron.* **8**(24) 10.1038/s41528-024-00307-1 (2024).

[26] Committee, C. Consensus statement on the definition of orthostatic hypotension, pure autonomic failure, and multiple system atrophy. The consensus committee of the American autonomic society and the American academy of neurology. *Neurology* **46**(5), 1470. 10.1212/wnl.46.5.1470 (1996). 10.1212/wnl.46.5.1470 8628505

[27] ...Mieloszyk, R. et al. A comparison of wearable tonometry, photoplethysmography, and electrocardiography for cuffless measurement of blood pressure in an ambulatory setting. *IEEE J. Biomed. Health Inform.* **26**(7), 2864–2875. 10.1109/JBHI.2022.3153259 (2022). 35201992 10.1109/JBHI.2022.3153259

[28] Cisnal, A. et al. Robust feature selection for BP estimation in multiple populations: towards cuffless ambulatory BP monitoring. *IEEE J. Biomed. Health Inform.* **28**(10), 5768–5779. 10.1109/JBHI.2024.3411693 (2024). 38857137 10.1109/JBHI.2024.3411693

[29] Makowski, D. et al. Neurokit2: a python toolbox for neurophysiological signal processing. *Behav. Res. Methods* **53**(4), 1689–1696. 10.3758/s13428-020-01516-y (2021). 33528817 10.3758/s13428-020-01516-y

[30] Taşcı, Karakuş, Çavuşoǧlu, D. & Akyön, F. BIOBSS: biological signal processing and feature extraction library (2024). https://github.com/obss/BIOBSS (accessed 02 May 2024).

[31] Zhang, Z.-M., Chen, S. & Liang, Y.-Z. Baseline correction using adaptive iteratively reweighted penalized least squares. *Analyst* **135**(5), 1138. 10.1039/b922045c (2010). 20419267 10.1039/b922045c

[32] Zhang, Z., Dong, J., Luo, X., Choi, K. & Wu, X. Heartbeat classification using disease-specific feature selection. *Comput. Biol. Med.* **46**(1), 79–89. 10.1016/j.compbiomed.2013.11.019 (2014). 24529208 10.1016/j.compbiomed.2013.11.019

[33] Christov, I. I. Real time electrocardiogram QRS detection using combined adaptive threshold. *Biomed. Eng. Online* **3**(1), 28. 10.1186/1475-925X-3-28 (2004). 15333132 10.1186/1475-925X-3-28 PMC516783

[34] Saenz-Cogollo, J.F. & Agelli, M. Investigating feature selection and random forests for inter-patient heartbeat classification. *Algorithms*. **13**(4) 10.3390/a13040075(2020).

[35] Mondéjar-Guerra, V., Novo, J., Rouco, J., Penedo, M. G. & Ortega, M. Heartbeat classification fusing temporal and morphological information of ECGS via ensemble of classifiers. *Biomed. Signal Process. Control* **47**, 41–48. 10.1016/j.bspc.2018.08.007 (2019).

[36] Zhang, K., Zhang, H., Li, S., Yang, C. & Sun, L. The pmemo dataset for music emotion recognition. In *Proceedings of the 2018 ACM on International Conference on Multimedia Retrieval. ICMR ’18*, 135–142. 10.1145/3206025.3206037 (Association for Computing Machinery, 2018).

[37] Choi, J.-H. & Loftness, V. Investigation of human body skin temperatures as a bio-signal to indicate overall thermal sensations. *Build. Environ.* **58**, 258–269. 10.1016/j.buildenv.2012.07.003 (2012).

[38] Lundberg, S.M. & Lee, S.-I. A unified approach to interpreting model predictions. In *Advances in Neural Information Processing Systems*, vol. 30 (eds. Guyon, I.) (Curran Associates, Inc., 2017).

[39] Keany, E. BorutaShap: a wrapper feature selection method which combines the Boruta feature selection algorithm with Shapley values. *Zenodo*10.5281/zenodo.4247618 (2020).

[40] Fuchs, B., Ejtehadi, M., Cisnal, A., Pannek, J., Scheel-Sailer, A., Riener, R., Eriks-Hoogland, I. & Paez-Granados, D. Detection of Autonomic Dysreflexia in Individuals With Spinal Cord Injury Using Multimodal Wearable Sensors. https://arxiv.org/abs/2508.03715 (2025).

